# Genuine participation in participant-centred research initiatives: the rhetoric and the potential reality

**DOI:** 10.1007/s12687-017-0342-4

**Published:** 2017-10-24

**Authors:** Oliver Feeney, Pascal Borry, Heike Felzmann, Lucia Galvagni, Ari Haukkala, Michele Loi, Salvör Nordal, Vojin Rakic, Brígida Riso, Sigrid Sterckx, Danya Vears

**Affiliations:** 1 Centre of Bioethical Research and Analysis, National University of Ireland (Galway), Galway, Republic of Ireland; 2 Centre for Biomedical Ethics and Law, Department of Public Health and Primary Care, KU Leuven, Leuven, Belgium; 3 Leuven Institute for Genomics and Society, KU Leuven, Leuven, Belgium; 4 Bruno Kessler Foundation, Trento, Italy; 5 Department of Social Research, University of Helsinki, Helsinki, Finland; 6 Institute for Biomedical Ethics and the History of Medicine and Department of Informatics, University of Zurich, Zurich, Switzerland; 7 Centre for Ethics, University of Iceland, Reykjavik, Iceland; 8 Center for the Study of Bioethics, University of Belgrade, Belgrade, Serbia; 9 Instituto Universitário de Lisboa (ISCTE-IUL), CIES-IUL, Lisbon, Portugal; 10 Bioethics Institute Ghent, Department of Philosophy & Moral Sciences, Ghent University, Ghent, Belgium

**Keywords:** Participant-centred research, ICT, Participatory engagement, Web 2.0

## Abstract

The introduction of Web 2.0 technology, along with a population increasingly proficient in Information and Communications Technology (ICT), coupled with the rapid advancements in genetic testing methods, has seen an increase in the presence of participant-centred research initiatives. Such initiatives, aided by the centrality of ICT interconnections, and the ethos they propound seem to further embody the ideal of increasing the participatory nature of research, beyond what might be possible in non-ICT contexts alone. However, the majority of such research seems to actualise a much narrower definition of ‘participation’—where it is merely the case that such research initiatives have increased contact with participants through ICT but are otherwise non-participatory in any important normative sense. Furthermore, the rhetoric of participant-centred initiatives tends to inflate this minimalist form of participation into something that it is not, i.e. something *genuinely* participatory, with greater connections with both the ICT-facilitated political contexts and the largely non-ICT participatory initiatives that have expanded in contemporary health and research contexts. In this paper, we highlight that genuine (ICT-based) ‘participation’ should enable a reasonable minimum threshold of participatory engagement through, at least, three central participatory elements: educative, sense of being involved and degree of control. While we agree with criticisms that, at present, genuine participation seems more rhetoric than reality, we believe that there is clear potential for a greater ICT-facilitated participatory engagement on all three participatory elements. We outline some practical steps such initiatives could take to further develop these elements and thereby their level of ICT-facilitated participatory engagement.

## Introduction

It has been argued that too many deliberations and decisions in health and genetic research contexts have focused predominantly on the perspectives of medical research professionals and seldom on the perspectives of the patient or research participant. In recent years, integrating this latter perspective in policy has been considered an important aspect for public health and research as part of a broader participatory approach. In this respect, Information and Communications Technology (ICT) may become a useful tool for reviving the democratic ideal of participatory engagement in fields such as health-related research through its potential to facilitate the reconstruction of hierarchal relationships to be more egalitarian. On the face of it, this trend seems to be reflected in participant-centred research (PCR) initiatives that are considered by some to place patients and research participants—particularly through use of ICT—increasingly at the centre of decision-making. In this paper, we consider whether, and to what degree, ICT-based PCR initiatives actually attempt a level of genuine ‘participation’, as it has been traditionally understood and as evident in the broader non-ICT research context, or if such research initiatives simply have increased contact with participants through ICT while being otherwise non-participatory in any important normative sense. We highlight that genuine ‘participation’ should enable a reasonable minimum threshold of participatory engagement through, at least, three central participatory elements: educative, sense of being involved and degree of control. While we would agree with criticisms that at present, in some well-known initiatives, genuine participation seems more rhetoric than reality, we believe there is clear potential for a greater ICT-facilitated participatory engagement on all three participatory elements. We outline some practical steps such initiatives could take to further develop these elements and thereby their level of ICT-facilitated participatory engagement.

## Contemporary health research: key features of a participatory approach

It has been argued that health-related discussions have focused overly on the perspectives of medical professionals, academics and politicians, often excluding the perspectives of the patient or non-expert (Mak et al. 2003). There have been calls for greater integration of citizens’ perspectives in public health policy as part of a broader participatory perspective (Richards et al. 2013). By ‘participatory’, we refer to the promotion of wider participation involving more non-experts in the decision-making process. This call is guided by the assumption that the public should, to some extent, participate in decisions regarding publicly funded and relevant services (Laird 1993; Morone and Kilbreth 2003) and that this will result in more representative and accountable policies (Litva et al., 2002). On a deeper level, the discursive or deliberative quality of participation is also important in order to free this process from dominating power relations and uninformed/irrational views and to involve a ‘transformation rather than simply the aggregation of preferences’ (Elster 1998: 3). It connects with important values, such as the rights and legitimate expectations regarding the relationship between the medical establishment and the people it serves.

Participatory medicine is considered today as a new possible evolution of medicine (Prainsack 2014) that will allow patients and citizens involved to be considered not just ‘lay’ people, but agents, or partners, who can expand knowledge and, together with experts, drive decisions regarding their healthcare future. This evolution has not been an overnight phenomenon. The *Alma Ata Declaration* (1978) indicates that people ‘have the right and duty to participate individually and collectively in the planning and implementation of their health care’ (art. IV).^1^ The WHO’s *Ottawa Charter for Health Promotion* (1986) in the section ‘Strengthen Community Actions’ regarding health promotion also states that health promotion implies ‘concrete and effective community action in setting priorities, making decisions, planning strategies and implementing them to achieve better health’ and recognises that ‘[a]t the heart of this process is the empowerment of communities, their ownership and control of their own endeavours and destinies’. Moreover, community development implies the use of ‘existing human and material resources in the community to enhance self-help and social support, and to develop flexible systems for strengthening public participation and direction of health matters’.^2^ Finally, the European Commission (2001) specifies the need to develop ‘horizontal relationships’ in both healthcare practices and relations, in order to allow better communication and use of medical knowledge.^3^ Some would say that this is nothing short of a patient revolution.^4^

One of the most commonly known methods to implement such participatory ideals is Fishkin’s ‘Deliberative Polling’ where a representative sample of the population is polled for their initial views on a particular issue, followed by a moderated and informed group discussion, and finally re-polled again where differences in responses are expected to arise (Fishkin and Luskin 2005; Fishkin 2003).^5^ Another key illustrative example of such a process can be seen with the use of a citizens’ jury—a form of participatory technology which reflects the intent to direct citizen engagement in the policy process (as opposed to the sole involvement of experts). A citizens’ jury method is composed of 12–24 randomly selected citizens, who are informed by several perspectives, often by experts referred to as ‘witnesses’. The jurors then go through a process of deliberation, and subgroups are often formed to focus on different aspects of the issue. Finally, the jurors produce a decision or provide recommendations in the form of a citizens’ report.

Such methods have been applied successfully in various fields, from pandemics, to human genetics, breast cancer screening, water management, water pollution and research priorities (Gooberman-Hill et al. 2008; Fish et al. 2014; Paul et al. 2008; Huitema et al. 2010; Braunack-Mayer et al. 2010; Bennett and Smith 2007; Anderson et al. 2011). Within the healthcare domain, public participation is increasingly integrated with the discourse of many health, science and political institutions (Kelty and Panofsky 2014; Wyatt et al. 2013).^6^ Such practices are adopted today in the therapeutic relationship (Emanuel and Emanuel 1992), in healthcare ethics committees (Moreno 1995) and for the so-called consensus conferences on relevant scientific, technological and social issues (GMOs, environmental issues, public health matters), in order to involve more people and a wider section of the population in building a shared decision on issues of public and common interest.

A large-scale exercise in deliberative democracy has been prominently used with regard to the British Columbia BioLibrary experience (Burgess 2014; O’Doherty et al. 2011; O’Doherty et al. 2012; Secko et al. 2009). In this particular case, lay people were invited randomly to participate. From the ones willing to participate, there were some chosen who could represent the region, using a stratification sampling technique. Then people came to the meeting place, where they had a previous information session, and were provided with a workbook and toolkit, since the method is especially useful when the public knowledge in the subject is low. As in democratic polling, they made smaller groups for discussion. The deliberation occurs between all the participants in a larger group. When it was not possible to raise a consensus, the situation was outlined, and the main opinions and reasons for disagreement were analysed and subjected to posterior reflection. Importantly, this has not just been a one-off, or sporadic, exercise and similar examples of deliberative community engagement can increasingly be noted in a global context, for instance, in the cases of the University of California biorepositories, the Mayo Clinic Biobank and community-based participatory research partnerships such as recently reported research involving Pacific Islanders as research collaborators (Dry et al. 2017; Olson et al. 2013; McElfish et al. 2017).

It is important to note throughout that deliberative and participatory practices continue to contain an essential element of expertise. Nevertheless, expert opinion is not a static concept. For instance, patients who suffer from certain diseases regularly become real experts on them. Hence, exchanges of experiences by patients are important types of expert opinions (e.g. see https://www.smartpatients.com ). The contribution of scientific experts plays a key role in this kind of process, also known as hybrid forum (Callon et al. 2001). Ultimately, such initiatives highlight that the value of traditionally designated experts is to be balanced with the wider relatively untapped expertise of the wider public (be it citizen, patient or research participant)—without, it should be noted, an ongoing role of the experts being removed.

## ICT-based participation: genuine ‘participation’ or merely increased non-participatory contact

We can ascertain a reasonably robust form of participatory engagement evident throughout such contemporary health research initiatives and one that has clear roots in both participatory and deliberative variants of a wider democratic theory. More than simply increasing participation in a numerical sense, the deliberative approach argues against a view of democracy that merely involves the aggregation of pre-existing and fixed preferences. Instead, they ascribe to an ideal where political decision-making involves free and equal citizens that listen to and respect each other, reasonably reflect on issues, give good reasons for their positions, seek to understand the perspectives of others and are willing to change their initial preferences during the process of deliberation (Burgess et al. 2008: 285). Moreover, such a practice is more deliberative the more it is a ‘process in which everyone concerned by the decision is considered as a valid moral agent, obliged to give reasons for their own points of view, and to listen to the reasons of others’ (Gracia 2003: 227). Importantly, deliberation should (ideally) have a ‘transformative’ effect, where it is not just a case of more ideas (even underrepresented ones) being added to the mix, but that the participants own ideas themselves will be developed and revised through engagement. It can be read as a form of dynamic consensus, because it implies ‘the self-discovery and transformation’ that are at stake in the consensus building processes (Moreno 1995: 71). In order to reach decisions that are acceptable to each participant, this mutual justification through informed and comprehensive reason-giving must occur within a context of mutual respect and reciprocity (Gutmann and Thompson 2002; Gracia 2003).

The overriding goal is to increase participation, especially in the form of influence (or even control) over important decision-making, and an influence that is not uninformed but that itself is the result of facilitated deliberation (Serdült and Welp 2015). In this sense, it can be understood as exemplifying an overall ideal of seeking to increase the ‘quantity of quality’ opportunities for citizens (i.e. patients or research participants) to be better involved within and to contribute to decision-making processes that affect their lives.^7^ Insofar as this is the case, the aforementioned forms of participatory initiatives would be examples of, what we call, *genuine* participatory engagement that can be increasingly seen in a non-ICT context. It is a plausible assumption, at least, that a greater use of ICT in facilitating such research relationships could further develop such participatory opportunities in a variety of emerging online contexts.

A good illustration of this can be seen where various participatory engagement initiatives integrating ICT exist in the broader political context where online-based interaction is supplementing (if not occasionally supplanting) traditional fora for political activity both in national and international contexts (Sonntagbauer et al. 2014). The executive summary of the United Nations Department of Economic and Social Affairs, 2014reviewed global progress in governmental use of ICT technology to create better interconnected, more transparent and responsive policies, in particular by allowing citizens to engage effectively in decision-making processes ‘[...] through decentralised governance” (2014: 89). In Brazil, some promising cases of ICT-based digital democracy can be observed, extending beyond limitations of more traditional off-line forms of discussions (Sampaio et al. 2011; Steibel and Estevez 2015; Mendonça 2015). While it would be difficult to practice a ‘physical’ participatory form of democracy in almost any contemporary state (as they are simply too big), and especially difficult to develop inclusive and competent deliberative processes within them, ICT can facilitate a number of interconnected, common interest-focussed groups without problems of geography and other factors limiting the traditional participatory scope. Indeed, Karlsson (2011) suggests that increasing numbers of participants in this new context may not weaken deliberation as might be expected in the traditional setting, but can strengthen it in novel ways (see also Manosevitch 2010).

With the introduction of Web 2.0 technology, an increasingly ICT-proficient population, and rapid improvements in genetic testing methods, there is also a growing presence of participant-centred research initiatives (Vayena and Tasioulas 2013; Kaye et al. 2012). It is not surprising that the growth of PCR initiatives has been promoted heavily by their apparent focus in strengthening participatory engagement and opting for increasingly egalitarian akin to a pure ‘citizen science’ context, as opposed to hierarchal, researcher-research subject/participant relationships (Woolley et al. 2016). Juengst et al. (2012) see the notion of patient empowerment as a central theme in their marketing as well as in the ‘enthusiastic writings of their customers’. Such initiatives are also considered by some ELSI commentators to place participants ‘at the centre of decision-making process’ (Kaye et al. 2012: 371). Participation can range from choosing which research projects participants want to be involved in (Kaye et al. 2012) to having a more substantial role in research initiatives where participants have the option to vote on which research topics are investigated or potentially self-initiate research projects (Weigmann 2014: 223). Indeed, Kelty and Panofsky (2014) have analysed the growing concept and practice of ‘participation’ across a number of science and medicine research initiatives, including such ICT-based participant-centred initiatives as PatientsLikeMe and 23andMe. In particular, they outline no less than seven dimensions that ‘participation’ can take including educative, relating to goals and tasks (i.e. participatory control over research goals), control over resources (e.g. the use of the data collected), right to exit research without undue cost, right to voice feedback and complaints, the use of visible metrics (enriching sense of participation) and the degree that participants can communicate with each other to produce affiliation and sociability. On the face of it, such initiatives, aided by the centrality of ICT interconnections, and the ethos they propound, seem to further embody, if not exemplify, the ideal of increasing the participatory nature of research, beyond what might be possible in non-ICT contexts alone.

However, as noted in Woolley et al. (2016), the vast majority of such participant-centred research can be seen to take place according to a much narrower definition of ‘participation’—where it is merely the case that such research initiatives have increased contact with participants through ICT but are otherwise non-participatory in any important normative sense of the word. While Kelty and Panofsky (2014) noted a large number of dimensions of participation, they also observed that the ICT-based PCR initiatives fared worse, in qualitative terms, than other earlier (non-ICT), and more purely, citizen science initiatives. For instance, Kelty and Panofsky (2014) note that in most cases, including 23andMe and PatientsLikeMe, the arguably most central defining characteristic of participation, that of control, was lacking. In the narrower understanding, more often than not, research participants have been seen as simply, ICT-facilitated ‘source of data gathered […] without being “engaged” or “involved” beyond informed consent’ (Woolley et al. 2016). The rhetoric of participant-centred initiatives, such as 23andMe or PatientsLikeMe (as well as, indirectly, in some of the ELSI responses), would seem to risk inflating this very minimalist form of participation into something that it is not, i.e. something *genuinely* participatory with greater connections with both the ICT-facilitated political contexts as well as the largely non-ICT participatory context outlined above.

## Genuine online participation—three necessary elements

Nevertheless, *assuming* that a broader, more genuine, form of participation is desirable, it is important to highlight how that can be actualised in platforms that would be readily useable by ICT-based PCR initiatives and also how its actualisation would be in keeping with improving the professed participatory goals of such initiatives.^8^ Simply, this stronger (more ‘genuine’) form of ICT-based participation could be actualised by strengthening a number of key participatory dimensions, such as those described as Kelty and Panofsky (2014). Before seeing how this is so, it should be evident that the seven dimensions can be grouped under three broader, but more clearly distinct, elemental categories—(a) an educative category, (b) a sense of being involved category and (c) a control category. The seven dimensions can be readily reducible as (1) ‘educative’ remaining as ‘educative’, (2) ‘visible metrics’ and ‘participants’ ability to communication with each other’ as ‘sense of being involved’ and (3) ‘control over goals and resources’, including ‘right to exit and voice opinion’ as ‘sense of control’. Our definition is not just simpler in formulation, but the three elemental categories better highlight the basic foundations underlying much of the theory and practice of participatory (and deliberative) approaches.

### The educative category

The educative dimension of participation realises the value of knowledge and insight, acknowledging their importance for individual reasoning and decision-making. It is common for participatory initiatives to employ educative components (Gutmann and Thompson, 2002). However, for educative components to contribute specifically to participation, rather than merely achieve an increase in knowledge, it is essential that information provision is closely linked to the interests of the participant (albeit subject to deliberative revision), rather than being primarily defined by the interests of the platform designers or other experts’ opinions on the relevance of information. New knowledge or insight in question should emerge at least partly through self-directed or shared engagement with the subject matter, rather than being based merely on top-down information delivery to participants.^9^

### The sense of being involved category

This dimension captures the value of membership of a community. Communities allow individuals to connect their own identity and endeavours with those of other persons and thereby situate themselves within a larger context (O’Neill 2006). Communities provide opportunities for mutual understanding, recognition, respect and support among members. The notion of *civic virtue* discussed by Kelty and Panofski (2014) which they include under ‘education’ might be more appropriate under this category. It allows the possibility of collective endeavours to achieve aims shared between its individual members including those living in very distant realities, countries and conditions. In this new digital context, ICT has the potential to enhance participation, including in key deliberative respects, where members of different communities are connected and can share common interests. In relation to health, these interests largely consist of patients receiving the best possible information and care. In research, the interests could be manifold—contributing to possible cures for themselves or others, advancing scientific knowledge, personal curiosity and so on. While many discussions of participation in political theory highlight the importance of equality, communities can allow asymmetric relationships while nevertheless being participatory, for example in facilitating relationships between experts and lay participants.

### The control category

Control, sometimes linked to the value of autonomy, allows a more direct and better command of, and over the data, the process and the results. Control that facilitates and fosters genuine participation requires involvement of the participants, not only in terms of acquisition of knowledge and awareness about specific issues, but also in terms of the possibility to express opinions, give advice, make complaints (and to have these heeded) and reach common decisions. As noted in Kelty and Panofsky (2014: 12), participants ‘want influence over goals, they want to share in the benefits of the resources created, they want genuine opportunities to engage scientists without too many barriers of expertise and authority’ (see also Yishai 2012).

In terms of assessing the participatory engagement of such PCR initiatives, for instance 23andMe and PatientsLikeMe, Kelty and Panofsky (2014) note normatively significant strengths in the educative and sense of being involved categories. In both these cases (and most cases they examine), the ‘control’ category is largely as Woolley et al.’s (2016) assessment would suggest, and this perhaps is the most important aspect of what would most crucially be genuinely participatory. However, it would also seem wrong to give the other two categories so little weight that they do not substantially affect the moral assessment of the overall participatory engagement of any given initiative. PCR initiatives that ‘score’ highly in these two aspects but not in the control aspect might not be the best, or ideal, form of participatory engagement, but they might be sufficient to be genuine forms of participation nonetheless. With due regard to the above criticisms (such as Woolley et al. 2016), it may be unfair to overly dismiss some genuine participatory aspects to some well-known ICT-based PCR initiatives, and perhaps better to note that there is a mix of rhetoric than reality involved. As Barbara Prainsack (2014) notes, it ‘is not *one* participatory medicine that universally empowers patients’ and the focus perhaps should be less on requiring a single PCR initiative to embody every participatory element to the fullest sense. Rather, the focus could be on greater interconnectivity between different PCR initiatives (and their ICT platforms) so that a given participant, with their data, can freely engage with a variety of initiatives, thereby increasing their overall participatory involvement. The question remains on how such participatory engagement (understood under all three categories) can be further developed in a way that is achievable through ICT platforms and conducive to the overall goals of PCR initiatives and is simple enough a model to be a guide accessible by relevant actors in the field. As an initial step in drafting such a model, we would propose something like the following list of ICT functionalities to enable participatory engagement along all three of the categories we outline above, in Table 1.^10^

**Table 1:** Description of functionalities required of platforms for each of the three participatory categories, educative, sense of involvement, and control

**Educative category**
***Functionalities:***
To be truly participatory, educative components should provide opportunities for participants to gain knowledge by means of some of the following functionalities:
Tailoring the provision of information to users’ interests and needs, with regard to what and how much information to receive
Elicitation of information or opinions from other participants, similar stakeholders or experts
Motivation of participants to increase their knowledge, e.g. through gamification
Testing of participants’ understanding
ICT supported informed consent that actively engages the participant in the process
Allowing return of results to participants
Allowing participants to provide feedback on the quality of information or their experience on the platform, e.g. in forms of ratings, endorsements, etc.
***Examples:***
Educative aspects of online platforms for participant-centred research could include information on a range of relevant topics, such as:
Specific conditions relevant to participants, e.g. as patients or family members
Reputable sources of information, including various stakeholder perspectives
Specific research projects, their study design and supporting information
Funding sources, e.g. involvement of commercial actors
Return of results
Impact of research
**Sense of being involved category**
*** Functionalities:***
To count as participatory, the platform should provide opportunities for participants’ engagement in a collective endeavour by means of some of the following functionalities:
Recruitment mechanisms that allow new participants to join a community
Provision and incentivization of interaction and sharing between participants
Specific barriers to participation addressed (e.g. language, literacy)
Facilitate active engagement between different groups of stakeholders, e.g. expert and lay members, around a common goal
Allow expression of appreciation to members or reward their community contributions
Facilitation of emotional engagement with the community
Facilitation of shared decision-making on goals and activities of the community
*** Examples:***
Community involvement elements could be represented through the following kinds of content provided on the platform:
Explicit definition of a specific community and/or valuable collective endeavour
Identification of participants as sharing an identity as members of such a community (understood in a wide sense, as only those who do/are X)
Provision of news on community activities and achievements
Provision of results from studies facilitated through the platform
Information on and facilitation of participation in fundraising or awareness raising initiatives
**Control category**
*** Functionalities:***
In order to realise the above forms of control, one would expect to see the following functionalities:
Ability to input into the design of platform
Ability for recruitment (expressions of interest for future studies, for example, sign up to a mailing list)
Ability to assist in design of research study (who participates; what are the measures; what is an outcome/goals)
Ability to control the results they receive
Ability to make decisions relating to study benefits, such as access to outcomes, exploitation of results, commercialisation
Ability to control over their level of participation, such as through different components, and decision-making ‘without penalty’
Ability to change inclusion/withdrawal status (dynamic consent)
*** Examples:***
The platform should provide users with opportunities to have genuine control over some of the following areas:
The goals or ends of the research, such as influencing the agenda, suggesting the outcomes that they desire or the questions they consider worthwhile exploring
The various means to achieve the research goals set by (expert) researchers, such as the way data should be collected/generated, participants to be included in the study
The use of data generated (by whom, in which contexts, for which purposes)
To have their voice heard, in terms of being able to complain and receive a response to such complaints, but also to make suggestions for improvements
To decide in an ongoing manner, such as through the use of dynamic consent, the various research directions the participants wish to be a part of

While the extent of participatory engagement underlying any ICT-based PCR initiative will depend on the relevant initiatives’ creators, it will also increasingly depend upon the demands of its participants. Furthermore, it is important to distinguish a related question of possibility or practicality of such a strengthened participatory endeavour. With regard to the potential strengths of increasing such participatory aspects, as indicated by the rhetoric used by such initiatives, as well as the examples from the non-ICT health research context and the emerging ICT-based political contexts, it is likely that substantial participatory engagement in this context may increasingly become more than rhetoric. Indeed, a recent study further highlights that such rhetoric ‘seems to have been endorsed by much of the mainstream genomics research community as a compatible extension of its own efforts’ (McGowan et al. 2017).

## Conclusion

It has been pointed out that the ‘lack of empowering patients and engaging them in their own health care cuts off the possibility for meaningful interactions and does a great disservice to selves as social product’ (Hester 2001, 42). As was in the traditional setting, so too would this apply in the emerging ICT-based context. In our preferred scenario, patients and citizens can become not just subjects for research and interventions, but instead partners in these processes. Nevertheless, throughout the above, we recognise that the process of participation needs to be structured and guided at some point by some experts, with them being scientists and/or ‘expert’ patients (De Schutter and Lenoble, 2010). This kind of dynamic and reciprocal process—to learn something together, to try to build solutions or find answers by letting them emerge from below—seems to be promising. What is particularly relevant is the idea and the reality of a community increasingly requiring online forms of engagement: with the recent globalisation processes, a community can be enlarged till the point of involving people living in very distant realities, countries and conditions. In this scenario, ICT has the potential to enhance participation (including deliberation) in a new digital context where members of different communities are connected and can share common interests. In relation to health, these interests largely consist of patients receiving the best possible information and care. In research, the interests could be manifold—contributing to possible cures for themselves or others, advancing scientific knowledge, personal curiosity and so on.

An important ongoing question to keep asking is whether they truly bring such benefits. Are these technologies involving all the people potentially interested? Which are the real or potential ‘vulnerable’ groups? What happens—in particular—for patients affected by rare and neglected diseases? Could they find any kind of testing or analysis pertinent for their condition? And what about the so-called digital divide, if we look forward a global healthcare improvement? At this stage, we should consider all these aspects in order to develop better participatory ICT supported systems, including a mix of commercial and publicly funded initiatives (Prainsack 2014). All these technologies—in their development and building process—can imply and represent forms of what Charles Sabel calls ‘democratic experimentalism’ (Sabel 2012): they can contribute and have a value also for understanding better how to improve participation and democratic processes for the healthcare field in our contemporary societies. In our opinion, a genuinely participatory approach can allow to improve a better knowledge and consciousness among people and scientists, in order to build a more effective ‘participatory’ medicine and to look forward in direction of new forms of participant-centred research initiatives and of a new ‘citizens science’.

## Footnotes

1 See http://www.who.int/publications/almaata_declaration_en.pdf

2 See http://www.euro.who.int/__data/assets/pdf_file/0004/129532/Ottawa_Charter.pdf

3 Commission of the European Community, *European Governance. A White Paper*, 2001

4
http://www.bmj.com/content/346/bmj.f2614; see also: https://genomethicsblog.org/2015/03/10/if-you-know-it-id-like-to-know-it-too-involving-participants-in-genomics-research/

5 For more information, see: http://cdd.stanford.edu/what-is-deliberative-polling/.This change in opinion turns should be carefully analysed to distinguish between an agreement and legitimation of expert opinions or a real deliberative process (Petersen 2007).

6 The necessity of arranging public dialogue is also emphasised as one of the 25 Recommendations that were provided by the STRATA group, invited in 2004 by the European Commission’s Research Directorate-General to give their recommendations related to the use of genetic testing in healthcare systems and genetic testing as a method of research (McNally and Cambon-Thomsen 2004). It is also mentioned in their recommendations that few data are available on ‘what children think’ and there is a need for further studies in this area. This objective is also in line with the European Union’s youth policy through its White Paper on A New Impetus for European Youth and the Communication from the Commission of 30 May 2005 on European policies concerning youth with the intention to promote active citizenship (European citizenship is also one of the priorities of the 2007 Youth in Action programme.).

7 This composite variant does not adhere to the more rigid classification present within democratic theory which notes tension between participatory and deliberative variants (Gutmann and Thompson 2002; Stokes 1998). It is not our purpose here to attempt to resolve this tension or to offer a perfect compromise between the two. Adopting the stronger, more robust composite variant we propose leaves it open to ways of resolving this tension or for either the participatory or deliberative side to be emphasised over the other (Blacksher 2013).

8 The desirability of a more genuine form of participation can be assumed to be a legitimate assumption, at least, in some cases (e.g. as noted above in cases of the wider health research contexts as well as suggested by the online political context in Latin America). In any case, this would be a separate argument than highlighting how it could be achieved, if desired.

9 The European Patients’ Academy (EUPATI) [www.eupati.eu] and the PlayDecide initiative [www.playdecide.eu] are two promising examples of educative engagement strategies. Our focus would be on both broadening the presence of such strategies throughout ICT-based PCR initiatives more generally and enabling an increase in the ICT-based interactions.

10 The lists of functionalities were created and revised over a number of working group meetings consisting of the co-authors and other working group members.
